# Propofol Suppresses Proinflammatory Cytokine Production by Increasing ABCA1 Expression via Mediation by the Long Noncoding RNA LOC286367

**DOI:** 10.1155/2018/8907143

**Published:** 2018-12-17

**Authors:** Xin Ma, Teng Wang, Zhen-Long Zhao, Yu Jiang, Shu Ye

**Affiliations:** ^1^Department of Anesthesiology, Nanfang Hospital, Southern Medical University, Guangzhou, China; ^2^Department of Cardiovascular Sciences, University of Leicester, Leicester, UK; ^3^Laboratory Medicine Center, Nanfang Hospital, Southern Medical University, Guangzhou, China; ^4^NIHR Leicester Biomedical Research Centre, Leicester, UK; ^5^Shantou University Medical College, Shantou, China

## Abstract

We previously reported that propofol upregulated the expression of ATP-binding cassette transporter subfamily A member 1 (ABCA1) via peroxisome proliferator-activated receptor gamma/liver X receptor in macrophage-derived foam cells. Here, we provide evidence that in addition to inducing ABCA1 expression, propofol represses proinflammatory cytokine production by increasing ABCA1 expression in a LOC286367-dependent manner. Western blot analysis showed that ABCA1 expression was elevated in macrophages by propofol treatment and this effect was markedly reduced by LOC286367 overexpression. Moreover, propofol treatment downregulated the production of the proinflammatory cytokines interleukin-6, tumor necrosis factor, and interferon gamma in lipopolysaccharide-stimulated macrophages by enhancing ABCA1 expression. Notably, propofol achieved this effect in a LOC286367-dependent manner. To the best of our knowledge, this is the first report of the mechanism in which propofol represses proinflammatory cytokine production mediated by ABCA1.

## 1. Introduction

Propofol (2,6-diisopropylphenol) is a well-established intravenous hypnotic agent that is pervasively exploited for the induction and maintenance of anesthesia as well as procedural sedation [1]. However, research with regard to the effects of propofol on inflammation is still in its infancy. It has been demonstrated that propofol exerts anti-inflammatory properties in septic patients [2] and in animal models [3–6]. While numerous literatures are emerging about the mechanisms of propofol's anti-inflammatory effects on sepsis of mice, such as through nuclear factor- (NF-) *κ*B pathways, knowledge about this process in human is fairly vague [4, 5]. A previous detailed analysis by our group concerning the effect of propofol on inflammation showed that propofol repressed lipopolysaccharide- (LPS-) induced expression of proinflammation cytokines via apolipoprotein M (apoM) in a hepatocyte nuclear factor 1 alpha-dependent fashion [7]. Moreover, propofol was found to decrease LPS-induced production of monocyte chemoattractant protein-1 (a cytokine that mediates cell influx to inflammatory sites) via increasing expression of apoM and forkhead box protein A2 (foxa2) in human liver carcinoma (HepG2) cells [8].

ATP-binding cassette transporter subfamily A member 1 (ABCA1) enhances unidirectional cholesterol efflux from macrophages onto apolipoprotein A1 (APOA1) (the major lipid-poor high-density lipoprotein (HDL) protein), initiating the process of reverse cholesterol transport (RCT). During this process, cholesterol is excreted into bile and feces, which is subsequently transported from peripheral tissues back to the liver through lymphatic and blood vessels [9–12]. In addition to the function of ABCA1 in cellular cholesterol homeostasis, a novel area of research has recently shed light on the aspects of its interaction with inflammation. Specifically, macrophage-specific deficiency of ABCA1 has been correlated with increased expression of proinflammatory genes and cytokine release in animal models [13]. Furthermore, deletion of ABCA1 from mouse macrophages led to cellular cholesterol accumulation, which has been associated with systemic inflammation and atherosclerotic lesion formation [14]. Intriguingly, similar findings were also observed in humans. Through a series of investigations of circulating monocytes as well as human monocyte-derived THP-1 cells, Bochem et al. [15] proposed that partial or total absence of ABCA1 was linked to a proinflammatory status in humans. These interesting findings provide evidence that ABCA1 exerts beneficial anti-inflammatory effects.

Long noncoding RNAs (lncRNAs) are transcripts of >200 nucleotides in length that are not translated into proteins and constitute a heterogeneous group of intergenic transcripts, enhancer RNAs, and sense or antisense transcripts that overlap other genes [16]. lncRNAs are transcribed from a vast number of genes, ranging from less than 20,000 to greater than 100,000 in humans [16–19]. Increasing evidence shows that lncRNAs serve as critical players in the expression patterns of various genes implicated in a vast number of cellular processes under both normal and pathological circumstances, such as cellular metabolism [20], bone metastasis [21], and colorectal cancer [22]. Of note, the role of lncRNAs in inflammation has recently attracted increasing attention due to the proinflammatory and anti-inflammatory functions of these transcripts in various diseases, including atherosclerosis [23] and rheumatoid arthritis [17]. Moreover, increasing lncRNAs have been identified as a mediator in anti-inflammatory process. For instance, lncRNA Lethe has been demonstrated to exert anti-inflammatory properties via repressing DNA binding of NF-*κ*B subunit RelA [24].

The results of the present study demonstrated that propofol enhanced ABCA1 expression by inhibiting lncRNA LOC286367 expression in THP-1 macrophages. Furthermore, LOC286367 and ABCA1 were involved in LPS-induced expression of inflammatory cytokines in response to propofol treatment. These results suggest that propofol suppresses the expression of proinflammatory cytokines by enhancing ABCA1 in a LOC286367-dependent manner. Interestingly, *in vivo* experiments showed that LOC286367 prompted an inflammatory response in LPS-treated BALB/c mice.

## 2. Results

### 2.1. Propofol Promotes ABCA1 Expression via Inhibiting lncRNA LOC286367 Expression in THP-1 Macrophages

To explore the effect of propofol on the expression of ABCA1 and lncRNA in THP-1 macrophages, we performed microarray analysis of lncRNA and mRNA differentially expressed in THP-1 macrophages treated with or without 50 *μ*M propofol. The results showed that expression of lncRNA LOC286367 was significantly decreased (3.62-fold, *p* = 0.0053) and that of ABCA1 was markedly increased (2.97-fold, *p* = 0.0015) in the propofol-treated groups, as compared to the control group (Figure S1).

Next, we investigated whether LOC286367 plays a role in the modulation of ABCA1 expression. By performing bioinformatic analysis of lncRNAs differentially expressed in THP-1 macrophages after propofol treatment that might target ABCA1 expression, we found that lncRNA LOC286367 (9q31.1, chr9: 107,536,633-107,540,045) and ABCA1 (9q31.1, chr9: 107,543,283-107, 690,527) were located on the same chromosome, but with opposite transcription directions (Figure S2). Pioneer studies on the characterization of lncRNAs have suggested that these molecules functionally modulate immediately neighboring genes, for instance, AIR [25] and Kcnq1ot [26]. Hence, these results indicated that LOC286367 probably plays a role in the regulation of ABCA1 expression.

**Figure 1:**
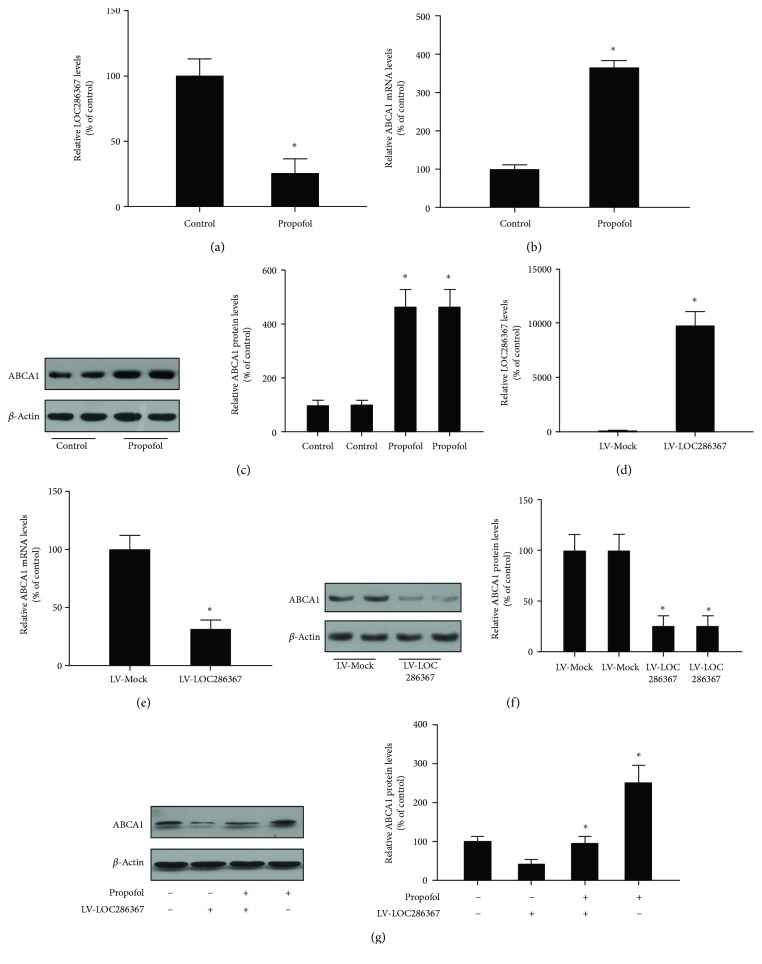
Effects of LOC286367 on propofol-induced upregulation of ABCA1 in THP-1 macrophages: (a–c) THP-1 macrophages were treated with or without 50 *μ*M of propofol for 24 h; (d–f) THP-1 macrophages were transfected with LV-Mock or LV-LOC286367; (g) THP-1 macrophages were transfected with LV-Mock or LV-LOC286367 and then incubated with or without 50 *μ*M of propofol for 24 h; (a, d, b, e) expression levels of LOC286367 ABCA1 mRNA were analyzed by qPCR; (c, f, g) ABCA1 protein levels were assessed by Western blot. All the results are expressed as mean ± SD from three independent experiments; each being performed in triplicate. ^∗^ *p* < 0.05 vs the control group.

Furthermore, we verified the results of microarray analysis by qPCR and Western blot assays. As shown, LOC286367 expression was significantly downregulated, whereas that of ABCA1 was markedly upregulated at both the mRNA and protein levels, as compared with the control group (Figures 1(a)–1(c), respectively).

To identify potential target candidates of lncRNA LOC286367, we selected an array of genes showing altered expression profiles according to results of microarray analysis. LV-Mock and LV-LOC286367 were created and transfected into THP-1 macrophages (Figure 1(d)). qPCR and western blot analyses were employed to measure the expression levels of candidate genes. As shown in Figures 1(e) and 1(f), overexpression of LOC286367 resulted in a significant reduction in both mRNA and protein levels of ABCA1.

We then explored the role of LOC286367 on propofol-induced ABCA1 expression. As shown in Figure 1(g), ABCA1 expression in THP-1 macrophages was enhanced by propofol treatment and this effect was completely abolished by LOC286367 overexpression. These results suggest that LOC286367 overexpression in propofol-treated THP-1 macrophages apparently suppresses ABCA1 expression.

### 2.2. LOC286367 and ABCA1 Are Involved in LPS-Induced Inflammatory Cytokine Production in Response to Propofol Treatment

**Figure 2:**
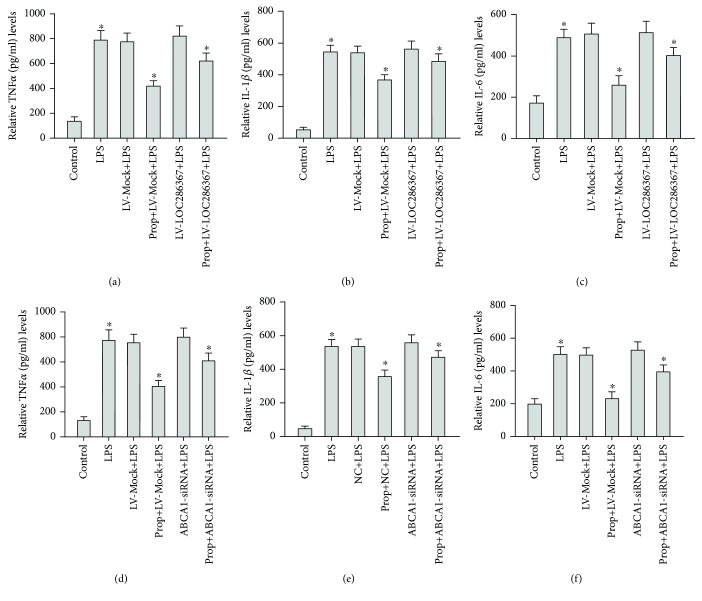
LOC286367 and ABCA1were involved in LPS-induced inflammatory cytokines with propofol treatment in THP-1 macrophage cells. (a–c) THP-1 macrophages were incubated with propofol (prop) (0 *μ*M), LPS (10 ng/ml), prop (0 *μ*M) + LV-Mock + LPS (10 ng/ml), prop (50 *μ*M) + LV-Mock + LPS (10 ng/ml), prop (0 *μ*M) + LV-LOC286367 + LPS (10 ng/ml), and prop (50 *μ*M) + LV-LOC286367 + LPS (10 ng/ml) in medium at 37°C for 24 h. (d–f) THP-1 macrophages were incubated with prop (0 *μ*M), LPS (10 ng/ml), prop (0 *μ*M) + negative control + LPS (10 ng/ml), prop (50 *μ*M) + negative control + LPS (10 ng/ml), prop (0 *μ*M) + ABCA1-siRNA + LPS (10 ng/ml), and prop (50 *μ*M) + ABCA1-siRNA + LPS (10 ng/ml) in medium at 37°C for 24 h. All results are presented as mean ± SD of the three independent experiments; each being performed in triplicate. ^a^ *p* < 0.05 vs the control group.

To explore the effect of LOC286367 on LPS-induced inflammatory cytokine production by propofol-treated THP-1 macrophages, we first examined the effects of LOC286367 overexpression on the expression levels of TNF-*α*, IL-1*β*, and IL-6 in propofol-treated, LPS-stimulated THP-1 macrophages. As shown in Figures 2(a)–2(c), propofol treatment remarkably downregulated the expression levels of TNF-*α*, IL-1*β*, and IL-6 and this effect was nearly reversed by LOC286367 overexpression in LPS-stimulated THP-1 macrophages. Overexpression of LOC286367 upregulated the expression levels of TNF-*α*, IL-1*β*, and IL-6 by 48%, 31%, and 55%, respectively. These results indicated that LOC286367 was involved in LPS-induced inflammatory cytokine production in propofol-treated THP-1 macrophages.

Next, we investigated the effect of ABCA1 on LPS-induced inflammatory cytokine production by propofol-treated THP-1 macrophages. Briefly, siRNA targeting ABCA1 efficiently inhibited ABCA1 expression. We observed the effects of ABCA1-siRNA on the expression patterns of TNF-*α*, IL-1*β*, and IL-6 in propofol-treated LPS-stimulated THP-1 macrophages. As shown in Figures 2(d)–2(f), propofol treatment remarkably downregulated expression of TNF-*α*, IL-1*β*, and IL-6 and this effect was nearly reversed by ABCA1-siRNA in LPS-stimulated THP-1 macrophages. ABCA1-siRNA enhanced the expression levels of TNF-*α*, IL-1*β*, and IL-6 by 50%, 32%, and 72%, respectively. These results indicated that ABCA1 was involved in LPS-induced inflammatory cytokine production by propofol-treated THP-1 macrophages.

### 2.3. LOC286367 Prompts Inflammatory Response in LPS-Treated BALB/c Mice

**Figure 3:**
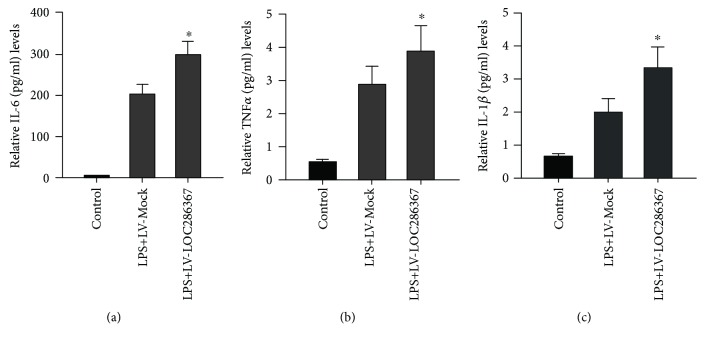
Effects of LOC286367 on inflammatory response in LPS-treated BALB/c mice. (a–c) BALB/c mice were randomly divided into three groups: group 1 received tail vein injection of LV-Mock (*n* = 6), group 2 received tail vein injection of LV-Mock and then LPS (250 *μ*g/kg) injection (*n* = 6), and group 3 received tail vein injection of LV-LOC286367 and then LPS injection (250 *μ*g/kg) (*n* = 6). Two hours after the injection, blood was harvested and ELISA was employed to measure the expression levels of IL-6, TNF-*α*, and IL-1*β* in serum. Values are presented as mean ± SD of three independent experiments. ^#^ *p* < 0.001 vs the control group.

Under normal physiological conditions, a great number of lncRNAs are expressed in an omnipresent fashion at a basal level across all human tissue types [27]. However, the expression levels of lncRNAs change during inflammation in diverse cell types. Thus, we explored the effect of LOC286367 on the inflammatory response in LPS-treated mice. Intriguingly, we found that LOC286367 possesses positive regulatory roles in the production of inflammatory cytokines. Packed empty LVs with LV-Mock and LV-LOC286367 were created and injected into the tail veins of mice (Figure 1(f)). Data on the expression level of IL-6, TNF-*α*, and IL-1*β* were collected at 2 h after administration. Western blot assessment showed that LV-LOC286367-transfected mice exhibited higher systemic levels of IL-6, TNF-*α*, and IL-1*β* in response to LPS treatment, as compared with the LV-Mock groups (Figures 3(a)–3(c)).

## 3. Discussion

ABCA1 prompts macrophages to efflux cholesterol onto APOA1—the major lipid-poor form of HDL protein—which is the first and crucial step in RCT [9–12]. Emerging evidence has shown that RCT is intimately associated with inflammation [28, 29]. ABCA1 also plays a significant role in the anti-inflammatory response through multiple pathways. For instance, inflammation represses ABCA1-mediated cholesterol efflux from macrophages by activating the Janus kinase/signal transducer and activator of the transcription pathway [30] and both signal transducer and activator of transcription 3 activation and cholesterol efflux are responsible for the anti-inflammatory effect of ABCA1 on macrophages [31]. Therefore, reduction of ABCA1 could cause a decrease in cholesterol efflux, leading to cholesterol accumulation and the promotion of inflammatory responses. ABCA1 may serve as a bridge linking cholesterol accumulation and activation of the inflammatory responses. Intriguingly, the correlation between cholesterol accumulation and inflammation is exemplified optically by atherosclerosis [32]. Thus, ABCA1 might act as a target for the prevention and treatment of atherosclerosis not only for its effects on cholesterol accumulation but also for its roles in the inflammatory response. Intriguingly, ABCA1 mutation carriers exhibit elevated systemic inflammation of plaque accumulation, which is dampened by statin treatment [15], suggesting that ABCA1-mediated anti-inflammatory effects are more intensive with drug treatment. Of note, our research is in agreement with the above proposal. In this study, microarray analysis demonstrated that propofol upregulated ABCA1 expression, which was confirmed by qPCR and Western blot analyses. Furthermore, ABCA1 is involved in LPS-induced inflammation with propofol treatment.

Our group previously found that lincRNA-DYNLRB2-2 expression was markedly induced by oxidized low-density lipoprotein, subsequent promotion of ABCA1-mediated cholesterol efflux, and repression of inflammation through G-protein-coupled receptor 119 in THP-1 macrophage-derived foam cells [33]. This finding provides evidence that lncRNAs play a regulatory role in ABCA1 expression. Pioneer research on the characterization of lncRNAs has suggested that these molecules are functionally regulated by neighboring genes [25, 26], broadly through several mechanisms, including promoter interference, chromosome remodeling, enhancer lncRNA, base paring with mRNA, miRNA precursors, and miRNA sponges. In addition, binding proteins change locations to modulate the activities of bound proteins [34]. Moreover, lncRNA-HC regulates ABCA1 by binding to its target mRNA hnRNPA2B1 [35]. Interestingly, bioinformatic analysis showed that lncRNA-LOC286367 neighbors ABCA1 and that the two molecules are located on the same chromosome with opposite transcription directions, indicating that LOC286367 might play a regulatory role in ABCA1 expression. Additionally, Western blot analysis results confirmed that LOC286367 apparently inhibited ABCA1 expression. However, the precise mechanism by which LOC286367 modulates ABCA1 is unclear; thus, further research is needed. The interaction of lncRNAs and inflammation has been extensively reported. For instance, the expression profile of lncRNA H19 is elevated in inflamed intestinal tissues of both mice and humans, which is induced by the inflammatory cytokine IL-22 [36]. The results of our *in vivo* experiments also suggested that LOC286367 exerts proinflammatory effects on LPS-treated mice. Mice with LOC286367 overexpression had higher systemic levels of IL-6, TNF-*α*, and IL-1*β* in response to LPS treatment, as compared with the LOC286367 deletion groups, which intensified the properties of LOC286367 in the inflammation response.

Recent *in vivo* and *in vitro* studies have shed light on the effects of propofol on the inflammatory response. Although the exact mechanism remains vague, some possible mechanisms have been proposed. For example, we previously demonstrated that propofol decreased expression of the LPS-stimulated monocyte chemoattractant protein-1 via increasing expression of apoM and foxa2 in HepG2 cells [8]. A recent experiment using the hippocampi of adult mice has revealed a mechanism of propofol in the regulation of lncRNAs. The study showed that propofol sedation can impact the expression of lncRNAs and mRNA in the hippocampus via the FoxO pathway-related proteins phosphatidylinositol 3-kinase and protein kinase B [37]. Intriguingly, the results of microarray analysis, qPCR, and Western blot assays performed in this study showed that propofol also exhibited an anti-inflammatory effect on THP-1 macrophages via inhibiting lncRNA-LOC286367 expression. Furthermore, propofol was reported to upregulate ABCA1 expression via peroxisome proliferator-activated receptor gamma/liver X receptor in THP-1 macrophage-derived foam cells [38]. However, the relationships among propofol, lncRNAs, and ABCA1 are unclear. Here, ABCA1 expression was increased in propofol-treated THP-1 macrophages and this effect was completely abolished by LOC286367 overexpression. Moreover, both ABCA1 and LOC286367 are involved in LPS-stimulated inflammatory responses with propofol treatment, further verifying that propofol regulated ABCA1 expression through LOC286367 during the inflammatory response in LPS-treated THP-1 macrophages.

In conclusion, the findings of this study suggest that propofol suppresses the proinflammatory response by reducing expression levels of proinflammatory cytokines, including IL-6, TNF-*α*, and IL-1*β*, in LPS-induced THP-1 macrophages via promoting ABCA1 expression, which is achieved in a LOC286367-dependent manner. These findings present a discovery platform for the relatively nascent area of lncRNA function in regard to the properties of propofol on inflammation. However, the link between propofol injection and ABCA1 expression remains to be elucidated. As research in this field forges ahead, the mechanism underlying how propofol interplays with ABCA1 in inflammation and novel mechanisms in addition to what we demonstrated here will likely emerge. Furthermore, broadening our understanding of the precise function of propofol in controlling the inflammatory response offers promising prospects for the development of new therapeutic strategies targeting a host of inflammation-related diseases, for instance, arthrosclerosis.

## 4. Materials and Methods

### 4.1. Materials

Propofol and LPS from *Escherichia coli* 055:B5 were purchased from Sigma-Aldrich Corporation (St. Louis, MO, USA). The PrimeScript RT Reagent Kit (Perfect Real Time; catalog no. DRR037A) and the SYBR Premix Ex Taq™ II Kit (Tli RNaseH Plus; no. DRR820A) were obtained from TaKaRa Bio Inc. (Shiga, Japan) as indicated before [7]. All other chemicals were of pharmaceutical grade and purchased from commercial suppliers.

### 4.2. Animals

Eight-week-old, female (more sensitive to inflammation than male ones [39]) BALB/c mice (Laboratory Animal Center of Guangdong, China) with a mean body mass of 20 g were randomized into three groups: group 1 received tail vein injections of mock lentivirus (LV-Mock) (*n* = 6), group 2 received tail vein injections of LV-Mock plus LPS (250 *μ*g/kg) (*n* = 6), and group 3 received tail vein injections of LV-LOC286367 plus LPS (250 *μ*g/kg) (*n* = 6). All animals were housed at five per cage at a constant temperature of 25°C under a 12 h light/dark cycle. The animal care and experimental procedures were approved by the Ethics Committee of Nanfang Hospital (Guangzhou, Guangdong, China).

### 4.3. Cell Culture

Human THP-1 macrophages were purchased from the American Type Culture Collection (Manassas, VA, USA) and maintained in Roswell Park Memorial Institute (RPMI) 1640 medium containing 10% fetal calf serum. After induction of differentiation for 72 h with 100 nM phorbol 12-myrustate 13-acetate (PMA) under standard culture conditions (5% CO_2_, 37°C), the cells were seeded in 6- or 12-well plates or 60 mm dishes and then differentiated into macrophages by the addition of 100 ng/ml of PMA for 72 h. Before the experiment, the cells were washed twice with phosphate-buffered saline and once with serum-free medium without antibiotics. The experimental media contained RPMI 1640 with 0.2% human serum albumin and one or more additives (i.e., LPS and propofol) at the concentrations described in the figure legends.

### 4.4. LncRNA Microarray Analysis

Total RNA was isolated using TRIzol reagent and then treated with DNase I (Invitrogen Corporation, Carlsbad, CA, USA) as demonstrated before [7]. Microarray analysis was performed on the Agilent Array platform. Briefly, mRNA was purified from 1 g of total RNA following removal of rRNA with an rRNA removal kit (Epicentre Biotechnologies, Madison, WI, USA). Next, each sample was amplified and transcribed into fluorescent cRNA along the total length of the transcripts without 3′ bias by utilizing a random priming method. The labeled cRNAs were hybridized onto the Human LncRNA Array v.2.0 (8960K; Arraystar Inc., Rockville, MD, USA). After extensively washing the slides, the Agilent Scanner G2505B was employed to scan the arrays. Agilent Feature Extraction Software v. 10.7.3.1 was employed to analyze the acquired array images. Then, quantile normalization and subsequent data processing were performed using the GeneSpring GX v11.5.1 software package (Agilent Technologies, Santa Clara, CA, USA). Hot map and hierarchical clustering were used to illustrate systematic variations in the differentially expressed lncRNAs and protein-coding RNAs among samples.

### 4.5. LV Production and Infection

Human monocytic THP-1 cells were cultured. Packed empty LVs with green fluorescent protein (LV-Mock) and LV-mediated lncRNA-LOC286367 overexpression vector (LV-LOC286367) were generated. The THP-1 cells were infected with the LV stock at a multiplicity of infection of 100 (THP-1 cells) transducing units per cell with 8 *μ*g/ml of polybrene to increase transduction efficiency. After incubation for 24 h, the cells were washed with fresh complete media.

### 4.6. RNA Isolation and Quantitative Real-Time PCR (qPCR)

Total RNA from cultured cells or human tissue was extracted with TRIzol reagent (TaKaRa Bio Inc.) in accordance with the manufacturer's instructions as indicated before [7]. The cDNA for lncRNA was synthesized with the All-in-One First-Strand cDNA Synthesis kit (GeneCopoeia, Rockville, MD, USA) in a 20 *μ*l reaction volume containing 1000–2000 ng of total RNA. The cDNA for mRNA was synthesized using the PrimeScript II 1st Strand cDNA Synthesis kit (TaKaRa Bio Inc.) in accordance with manufacturer's instructions. qPCR was performed using a SYBR Green PCR kit (TaKaRa Bio Inc.) and an ABI 7500 Fast Real-Time PCR System (Applied Biosystems, Foster City, CA, USA). U6 and glyceraldehyde 3-phosphate dehydrogenase were employed as internal controls for lncRNA and mRNA, respectively. The expression profiles were quantitated with the 2^−ΔΔCT^method. All samples were prepared in triplicate, and the mean value was used for comparative analyses. Primers were used as follows: ABCA1 forward, 5′-GTCCTCTTTCCCGCATTATCTGG-3′; ABCA1 reverse, 5′-AGTTCCTGGAAGGTCTTGTTCAC-3′.

### 4.7. Western Blot Analyses

Proteins were extracted from mouse tissues or cultured cells using RIPA buffer (Biocolor Ltd., Belfast, Northern Ireland, UK) as mentioned before [7]. The expression levels of proteins were quantified with ONE software (Bio-Rad Laboratories, Hercules, CA, USA) and normalized to that of *β*-actin. Then, the proteins were subjected to Western blot analyses (10% sodium dodecyl sulfate-polyacrylamide gel electrophoresis; 50 *μ*g protein per lane) employing rabbit anti-ABCA1 (Novus Biologicals, Littleton, CO, USA).

### 4.8. Measurement of Cytokine Production

THP-1 cells were differentiated into macrophages with 100 nM PMA for 72 h and then incubated with or without LPS in the absence or presence of propofol as indicated before [7]. After stimulation and sample treatment, the cell-free supernatants of the THP-1 macrophages were collected and used for analysis of cytokine production. The expression profiles of tumor necrosis factor- (TNF-) *α*, interleukin-1*β* (IL-1*β*), and IL-6 were measured in duplicate using a Biotrak™ enzyme-linked immunosorbent assay kit (Amersham Biosciences Corporation, Piscataway, NJ, USA).

### 4.9. Transfection with Small Interfering RNA (siRNA)

The siRNA against ABCA1 was purchased from Ribo Biotech Co. Ltd. (Guangzhou, Guangdong, China). THP-1 macrophage-derived foam cells (2 × 10^6^ cells/well) were transfected using Lipofectamine 2000 (Invitrogen Corporation). At 48 h after transfection, qPCR and Western blot assays were performed.

### 4.10. Statistical Analyses

Statistical analyses were performed using IBM SPSS Statistics for Windows, version 20.0 (IBM Corporation, Armonk, NY, USA) and GraphPad software v.6.0 (GraphPad Software Inc., La Jolla, CA, USA). Data are expressed as the means ± standard deviation. Results were analyzed by one-way analysis of variance followed by the Student–Newman–Keuls test. SPSS v13.0 statistical software (SPSS Inc, Chicago, IL, USA) was used to perform the two-tailed unpaired *t*-test. A probability (*p*) value of <0.5 was considered statistically significant.

### 4.11. Study Highlights

The anaesthetic drug propofol has recently attracted great attention for its anti-inflammatory effects. ABCA1 is well known for maintaining cholesterol homeostasis; however, its anti-inflammatory properties have been a novel highlight to explore. We previously demonstrated that propofol could upregulated ABCA1 expression. Moreover, lncRNAs play a crucial role in inflammatory response. We and others have suggested that lncRNAs exert a role in regulating ABCA1 expression. Additionally, propofol could impact the expression of lncRNA. However, the relationship among propofol, ABCA1, and lncRNA remains unknown. In this study, we showed that propofol inhibits proinflammatory cytokine production by increasing ABCA1 expression via mediation by lncRNA-LOC286367. To the best of our knowledge, this is the first study that provides systematically the mechanistic explanation for propofol-regulated ABCA1 expression during inflammatory response. Considering the anti-inflammatory property of propofol, presumably, it is promising to use propofol for treating inflammation-related diseases, such as atherosclerosis.

## Data Availability

The data used to support the findings of this study are available from the corresponding author upon request.

## Supplementary Materials

Supplementary 1 Fig.  S1: hierarchical clustering for the levels of ABCA1 and lncRNA with or without 50 *μ*M propofol. Hierarchical clustering based on the selected set [393 mRNAs (ABCA1 included) and 265 lncRNAs (LOC286367 included)] (red: upregulation; green: downregulation; black: no significant change).

Supplementary 2 Figure S2: diagram about the genomic locations and opposite transcriptional directions of LOC286367 and ABCA1.
