# Supplementary figures and images for: Propofol Suppresses Proinflammatory Cytokine Production by Increasing ABCA1 Expression via Mediation by the Long Noncoding RNA LOC286367
Source: Mediators Inflamm. 2018 Dec 17;2018:8907143. doi: 10.1155/2018/8907143 (PMC6311839; doi:10.1155/2018/8907143)

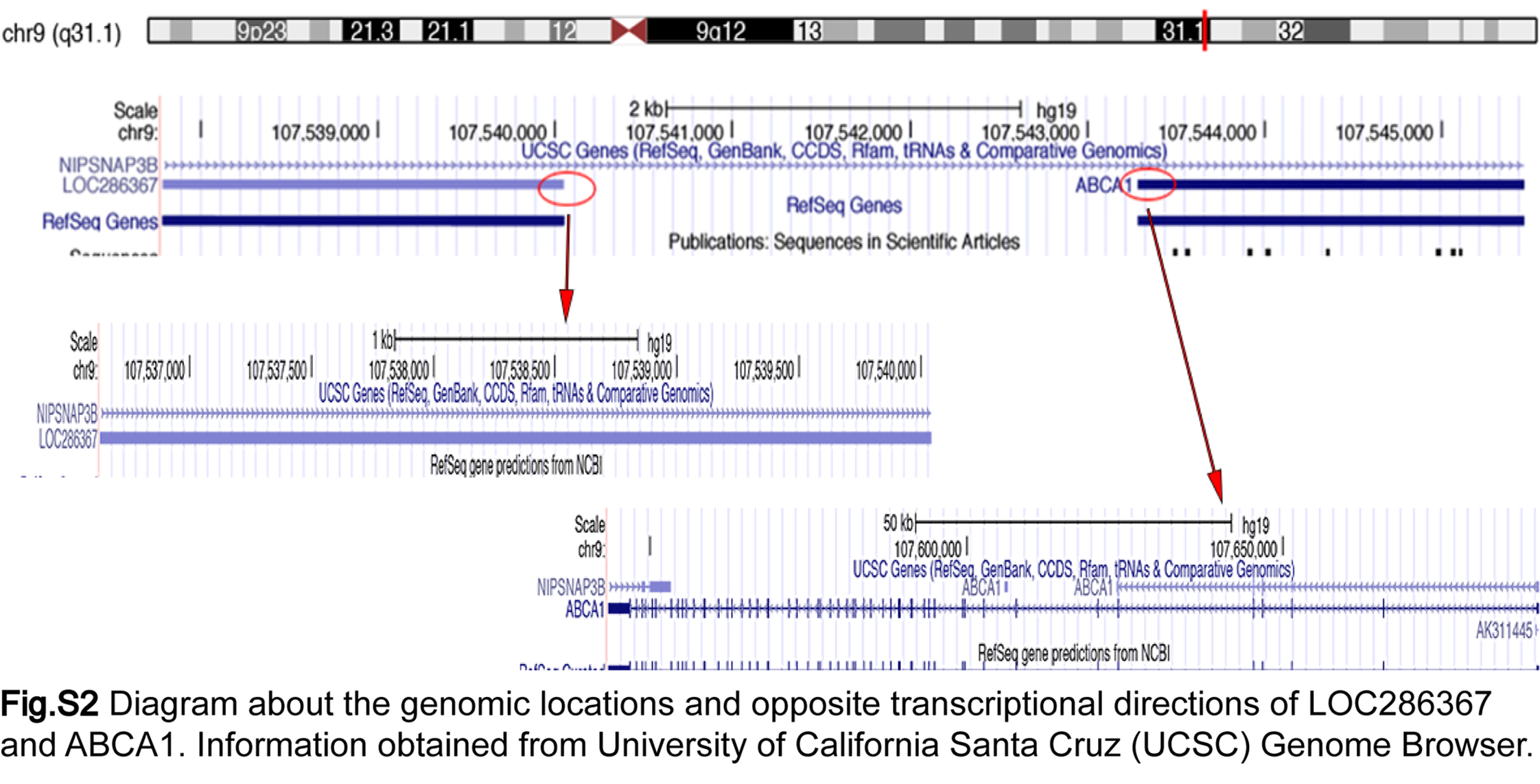

Supplement: Supplementary 1 — Fig. S1: hierarchical clustering for the levels of ABCA1 and lncRNA with or without 50 μM propofol. Hierarchical clustering based on the selected set [393 mRNAs (ABCA1 included) and 265 lncRNAs (LOC286367 included)] (red: upregulation; green: downregulation; black: no significant change). [file 8907143.f1.tif]

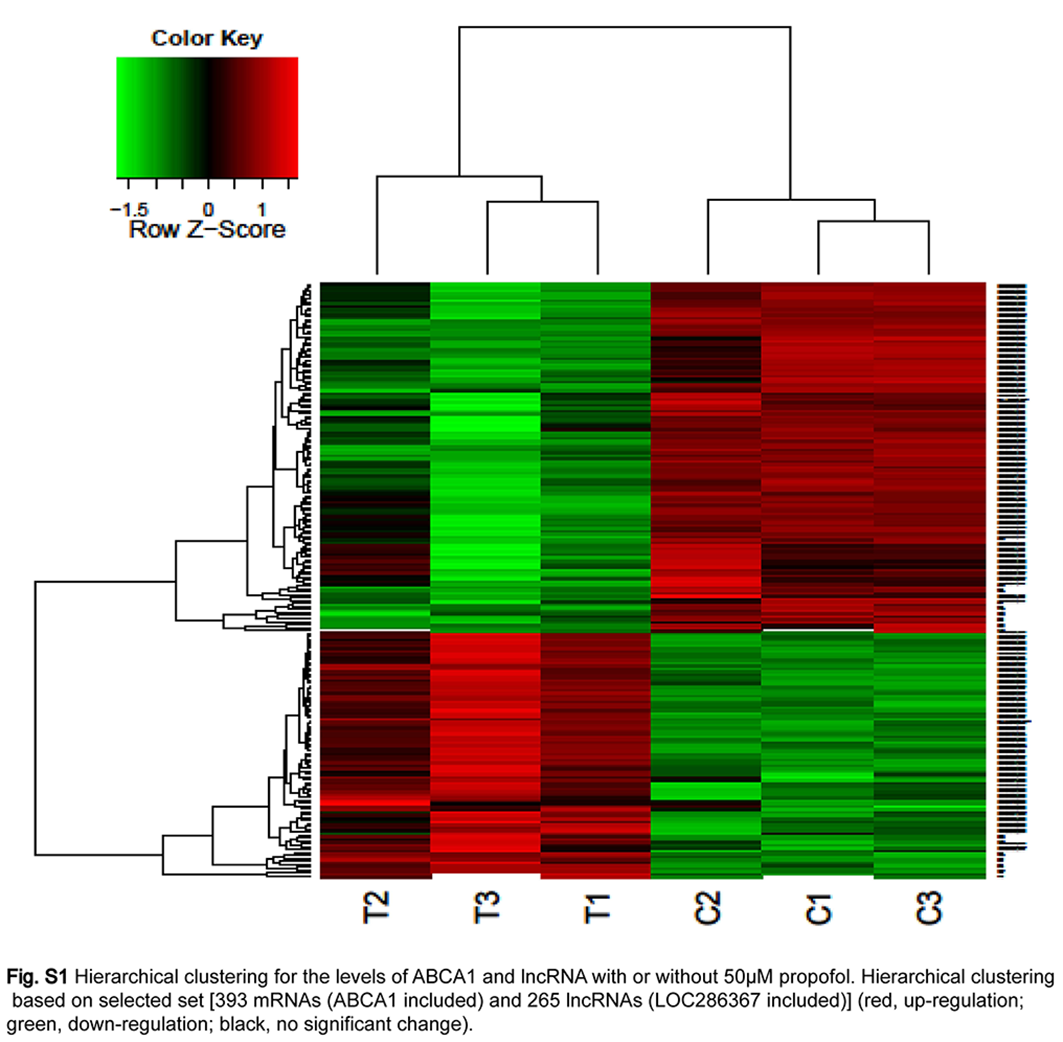

Supplement: Supplementary 2 — Figure S2: diagram about the genomic locations and opposite transcriptional directions of LOC286367 and ABCA1. [file 8907143.f2.tif]
